# Assessing Stages of Objective Memory Impairment and neuroimaging as risk factors of incident cognitive impairment

**DOI:** 10.1017/S1355617725101240

**Published:** 2025-08-22

**Authors:** Kellen K. Petersen, Ali Ezzati, Bhargav T. Nallapu, Richard B. Lipton, Reisa A. Sperling, Kathryn V. Papp, Dorene M. Rentz, Keith A. Johnson, Ellen Grober

**Affiliations:** 1 Department of Neurology, Albert Einstein College of Medicine, New York City, NY, USA; 2 Department of Neurology, Washington University in St Louis, St Louis, MO, USA; 3 Department of Neurology, University of California, Irvine, CA, USA; 4 Department of Cognitive Robotics, Technical University of Delft, Delft, The Netherlands; 5 Brigham and Women’s Hospital, Boston, MA, USA; 6 Massachusetts General Hospital, Harvard Medical School, Boston, MA, USA

**Keywords:** Free and Cued Selective Reminding Test, memory, incident cognitive impairment, biomarkers, entorhinal corex, Stages of Objective Memory Impairment

## Abstract

**Objective:**

The Stages of Objective Memory Impairment (SOMI) system, based on the Free and Cued Selective Reminding Test (FCSRT), is a potential marker of subtle cognitive impairment in cognitively normal persons defined by a Clinical Dementia Rating (CDR) = 0. We investigated SOMI’s ability to predict incident cognitive impairment (CDR >0) in combination with demographic features and neuroimaging biomarkers.

**Methods:**

Cognitively unimpaired participants (CDR = 0) from the Harvard Aging Brain Study had baseline FCSRT scores, MRI, FDG-PET, and PiB-PET as well as follow-up CDRs for 5 years. Cox proportional hazards models with correction for multiple testing assessed the predictive validity of SOMI and neuroimaging biomarkers for progression (CDR >0). Comprehensive sensitivity analyses examined alternative outcomes and stricter screening criteria.

**Results:**

Participants (*N* = 231) were 73.7 years (SD = 6.0), 60.2% were female, 29.0% were APOE4 positive, and 54 (23.4%) progressed to CDR >0. At baseline, 67% were SOMI-0, 22% were SOMI-1, 4% were SOMI-2, and 7% were SOMI-3/4. After multiple testing correction, hazard ratios (HRs) using SOMI-0 as reference were: SOMI-1 = 2.06 (CI: 1.09 – 3.88), SOMI-2 = 2.85 (CI: 1.08 – 7.54), and SOMI-3/4 = 3.73 (CI: 1.58 – 8.79, *p* = 0.016). SOMI-3/4 remained significant across most biomarker models. Entorhinal thickness emerged as the most robust biomarker predictor (HR = 0.57 – 0.65, *p* ≤ 0.015). Sensitivity analyses confirmed robustness across alternative outcomes and stricter screening criteria.

**Conclusions:**

SOMI stages predict progression to incident cognitive impairment with SOMI-3/4 maintaining significance after rigorous multiple testing correction. Entorhinal thickness provides the strongest biomarker enhancement to prediction models. SOMI demonstrates substantial incremental predictive value beyond standard demographic and biomarker predictors.

## Introduction

Alzheimer’s disease (AD) is a progressive, neurodegenerative disorder with a long asymptomatic preclinical phase of pathologic and pathophysiological changes. (Rafii and Aisen, 2023, Scharre, 2019). The hallmark cognitive deficit of AD is episodic memory impairment which emerges early in the AD continuum (Grober et al., 2000). The earliest site of AD neuropathology is the entorhinal cortex (Braak and Braak, 1991, Maass et al., 2018), pathology which is associated with memory decline

(Xie et al., 2025). Herein, we assess the utility of these two features in identifying cognitively unimpaired seniors at greatest risk of progression to symptomatic cognitive impairment in the Harvard Aging Brain Study (HABS) cohort. In this cohort we previously demonstrated an association between the Stages of Objective Memory Impairment (SOMI) system and structural and biofluid markers of amyloid and tau, the two neurotoxic proteins in AD (Jack et al., 2024).

SOMI defines five stages of episodic memory impairment beginning in preclinical AD using free recall (FR) and total recall (TR) scores from the Free and Cued Selective Reminding Test (FCSRT) to classify participants. In SOMI-0, FR and TR are normal. FR declines in the next two SOMI stages (SOMI −1,−2) reflecting increasing difficulty retrieving stored memory in the context of intact TR. Cuing fails to recover all the missed items in FR in SOMI-3 and SOMI −4 indicating an impairment of memory storage. In our previous report from the HABS study, we found that individuals in SOMI-3 or −4 had higher entorhinal and inferior temporal tau burden as well as smaller hippocampal volumes than individuals in SOMI-0 or −1 (Grober et al., 2021).

The current study was designed to test how well memory deficits defined by SOMI and neuropathology in the entorhinal cortex predict incident cognitive impairment among individuals categorized as cognitively unimpaired based on a Clinical Dementia Rating (CDR) of 0 at enrollment (Morris, 1993). We defined symptomatic cognitive impairment by a CDR >0, where CDR >0 includes both mild cognitive (MCI) impairment and very early dementia (Burke et al., 1988, McCulla et al., 1989, Morris et al., 2001, Morris et al., 1997). Based on previous findings, we hypothesized that 1) higher SOMI stages at baseline would be progressively associated with an increased risk of incident cognitive impairment; 2) neuroimaging measures related to the entorhinal cortex would be significant predictors of progression; and 3) SOMI would retain its predictive ability with the inclusion of neuroimaging biomarkers of amyloid, hippocampal volume, cortical thickness, and glucose metabolism.

## Methods

### Participants

Eligible participants were enrolled in the HABS, were cognitively unimpaired at baseline with a global CDR score of 0 at enrollment. Inclusion criteria for HABS were 65 years of age or older, scores above age- and education-adjusted cutoffs on the 30-minutes Delayed Recall portion of the Logical Memory Story A, a score of less than 11 on the Geriatric Depression Scale (GDS), and a score of 25 or greater on the Mini-Mental State Examination (MMSE) (Dagley et al., 2017). Exclusion criteria included a history of alcoholism, drug abuse, head trauma, or current serious medical or psychiatric illness. These inclusion and exclusion criteria ensured that the HABS cohort was comprised of initially cognitively unimpaired, healthy older adults when enrolled in the study (Dagley et al., 2017).

**Figure 1.**
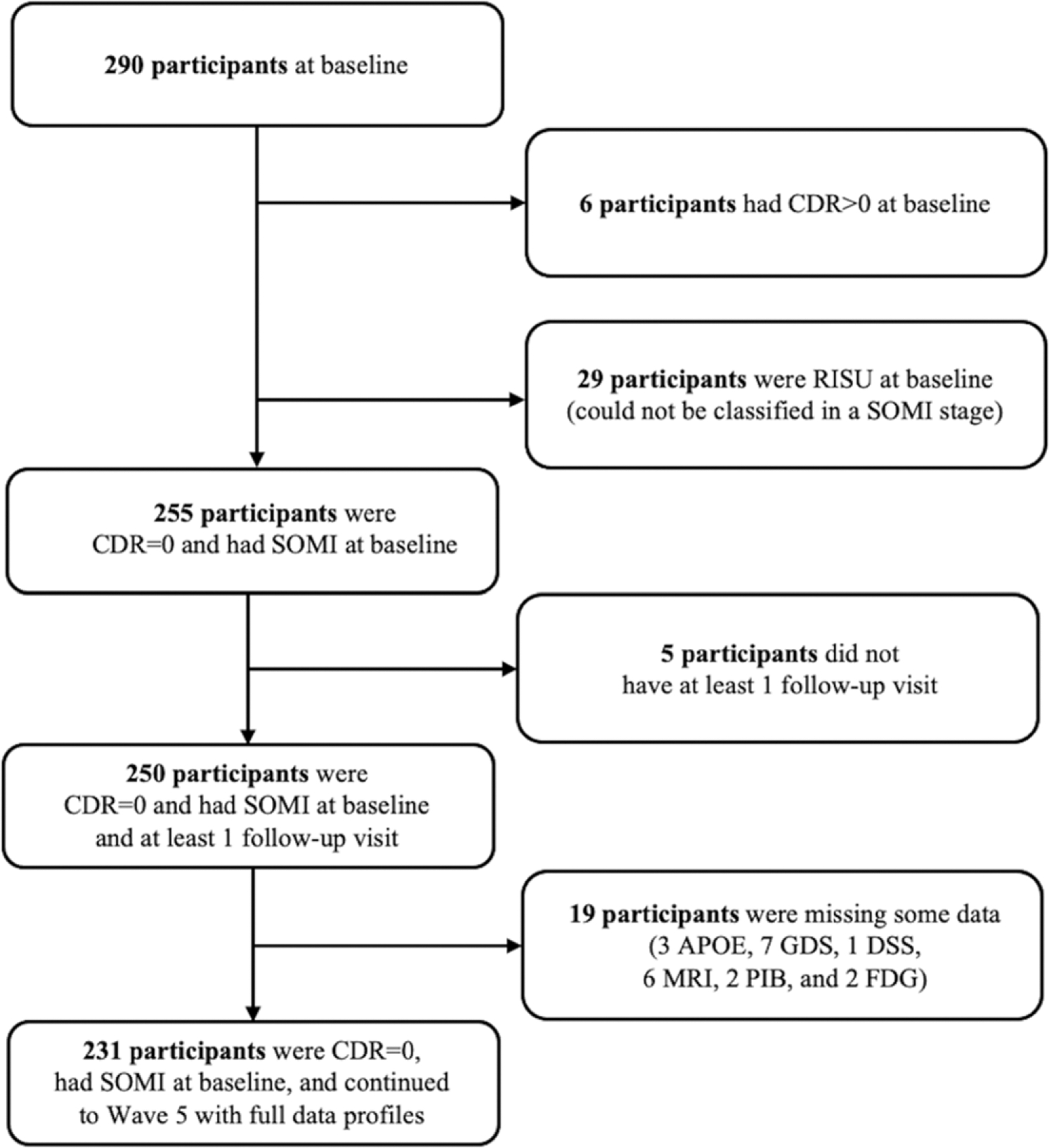
Flow diagram used to define the eligible sample. *Notes*. Abbreviations: CDR = Clinical Dementia Rating, SOMI = Stages of Objective Memory Impairment, RISU = Retrieval Impaired Storage Unimpaired (participants do not fit into the SOMI staging system), APOE = apolipoprotein genotype, GDS = Geriatric Depression Scale, DSS = Digit Symbol Substitution, MRI = magnetic resonance imaging, PIB = Pittsburgh compound B positron emission tomography, FDG = 18 F-fluorodeoxyglucose positron emission tomography.

The sample selected for this study was 231 HABS participants who had baseline FCSRT scores, longitudinal CDR measures, and neuroimaging biomarker data for volumetric magnetic resonance imaging (MRI), ^18^F fluorodeoxyglucose (FDG) PET, and ^11^C Pittsburgh Compound B (PiB) PET. A flow chart of study participants is shown in Figure 1.

### Standard protocol approvals, registrations, and patient consents

Study protocols for the use of human participants in the study were approved by the Mass General Brigham (Partners) Human Research Committee and written informed consent was obtained from all participants.

#### Clinical and neuropsychological assessments

HABS collects a variety of demographic information including age, sex, education, and race as well as APOE genotyping. Additionally, participants undergo annual clinical (e.g., CDR, MMSE, and GDS) and neuropsychological assessments. The CDR is assessed by neuropsychologists and psychiatrists without access to other cognitive test results (Papp et al., 2020). CDR raters make determinations blinded to participant biomarker status with quarterly consensus meetings conducted by 6 or more clinicians.

The picture version of the FCSRT with Immediate Recall (pFCSRT + IR) (Grober and Buschke, 1987) was administered as part of the neuropsychological assessment. In the encoding phase, participants identify pictured items (e.g., grapes) in response to category cues (e.g., fruit) that are used in the test phase to prompt recall of items not retrieved by free recall. Scores include free recall (FR: range 0–48) and total recall (TR: range 0 – 48). Using score ranges of FR and TR, participants were stratified into SOMI stages as shown in Table 1. Twenty-nine individuals, whose retrieval was impaired (FR <20), but storage was unimpaired (TR >46; RISU) at baseline, could not be classified by the SOMI staging system and were not included in the analysis. Due to the small number of participants in SOMI-3 and −4 stages, SOMI-3 and −4 were combined and treated as a single group (SOMI-3/4).

#### Imaging biomarkers

Volumetric MRI. Three-dimensional structural T1-weighted MRI scans were acquired with a Siemens Tim Trio 3T System with a 12-channel head coil (Siemens, Erlangen, Germany). Structural T1-weighted volumetric magnetization-prepared, rapid acquisition gradient echo (MPRAGE) scans were collected. Regions of interest (ROI) were identified and labeled using FreeSurfer software (version 6.0) with the Desikan-Killiany parcellation atlas (Desikan et al., 2006). Cortical thickness measures were obtained for the ROIs. Entorhinal and inferior temporal cortices were the regions of interest as in other HABS studies. Hippocampal volume (HV) was summed across hemispheres and adjusted (HVa). HVa was calculated to account for differences in intracranial volume using a residual regression approach (Hampton et al., 2023, Mormino et al., 2014a).

PiB- and FDG-PET. All PET data were obtained using a Siemens/CTI ECAT HR Scanner (3D mode; 63 image planes; 15.2 cm axial field of view; 5.6 mm transaxial resolution; 2.4 mm slice interval). Before injection, 10-minute transmission scans for attenuation correction were collected. PET data were reconstructed, attenuation corrected, and then manually evaluated to verify adequate count statistics and to correct for motion. After injection of 8.5 – 15 mCi PiB, 60-minutes of dynamic data were acquired in 3D acquisition mode and reconstructed in 39 frames (8×15s, 4×60s, and 27×120s). FDG was acquired from 45 to 75 minutes after a 5 – 10 mCi bolus injection and reconstructed into a single 30 minute frame (Dagley et al., 2017). For PiB-PET analysis, a global composite standardized uptake value ratios (SUVR) was calculated as the mean across frontal, lateral temporal, parietal, and retrosplenial regions, normalized to cerebellar gray matter reference region. For FDG-PET analysis, regional SUVRs were calculated for four regions of interest: entorhinal cortex, inferior temporal cortex, hippocampus, and isthmus cingulate cortex, normalized to pons reference region consistent with established protocols in aging and AD research. These FDG regions were selected based on their established role as early sites of AD pathology and metabolic dysfunction, consistent with prior HABS publications (Mayblyum et al., 2021).

#### APOE4 genotyping

To assess the effects of Apolipoprotein ε4 (APOE4) genotype in this analysis, we dichotomized APOE genotype. Individuals who possessed at least one copy of the APOE ε4 allele were categorized as APOE4 + , while others were classified as APOE4−.

#### Statistical analysis

MATLAB (version 2021a) was used to analyze data. Statistical significance was established at *α* = 0.05, and all tests were two-tailed. For sample characteristics among SOMI groups and the overall cohorts, continuous variables were examined using analysis of variance (ANOVA), and categorical variables underwent χ^2^ tests. Post hoc analysis was performed using Fisher’s least significant difference test.

Cox proportional hazards models were used to assess the association of SOMI stage and incident cognitive impairment, defined by the transition of CDR score from 0 at baseline to >0 during follow-up. Data from study participants who did not show a change in CDR over the course of follow-up were right-censored at the date of their last clinical assessment. SOMI stage was treated as a categorical variable with SOMI-0 as the reference stage. Age and APOE4 status were included in all models.

Seven models were developed to systematically evaluate the predictive utility of SOMI stages in combination with neuroimaging biomarkers. Model 1 included only demographic variables and APOE4 status as covariates. Models 2 and 3 added single biomarker measures (PiB-PET global SUVR and adjusted hippocampal volume, respectively). Model 4 incorporated cortical thickness measures (entorhinal and inferior temporal). Model 5 added FDG-PET measures (entorhinal, inferior temporal, and hippocampal). Model 6 combined cortical thickness and FDG-PET measures. Model 7 focused on the most predictive biomarker combination (entorhinal thickness, PiB-PET, hippocampal volume, and entorhinal FDG-PET).

Given the relatively low events-per-variable ratio (6.0 – 10.8 across models), we applied Benjamini-Hochberg correction for multiple testing across statistical tests to control the false discovery rate at 5%. This conservative approach addresses concerns about Type I error inflation while maintaining adequate power for detecting genuine associations. Events-per-variable ratios were calculated and reported for each model to ensure transparency regarding statistical power limitations.

The Maddala-Magee index, a measure of explained variation derived as an R^2^ measure based on the likelihood ratio test statistic applied to Cox proportional hazards regression, was calculated for each model. Partial likelihood ratio tests were performed between nested models to assess model improvement as biomarkers were added. Kaplan-Meier survival curves were used to illustrate survival probability over time.

Additionally, we conducted two sensitivity analyses to evaluate the robustness of our findings and address potential baseline classification bias. First, we examined an alternative outcome definition using CDR-SB ≥1 instead of CDR >0 to test whether findings persisted with a more stringent cognitive outcome measure. Second, we tested findings under stricter baseline cognitive screening criteria by restricting analyses to participants performing above one or two standard deviations of age- and education-adjusted norms on all neuropsychological tests (MMSE, FCSRT, Logical Memory, and Trail Making Tests). Age- and education-adjusted z-scores were calculated using linear regression models fitted to the baseline sample, with residuals standardized to create normative scores. Both sensitivity analyses used identical statistical methods as the primary analysis, including the same covariates, multiple testing correction procedures, and 5-year censoring approach, to ensure comparability of results.

## Results

### Sample Characteristics

A summary of baseline characteristics for the entire cohort as well as subsets stratified by changes in CDR status, is presented in Table 2. Participants were, on average, 73.7 (SD = 6.0, range 68.5 – 89.3) years old, had 15.9 (2.9) years of education, 60.2% were female, 81.8% were White, and 29.0% were APOE4+. Participants were censored at 5 years with a mean follow-up time of 4.06 (1.39) years. During follow-up, 54 (23.4%) participants developed incident cognitive impairment and 177 (76.6%) remained cognitively unimpaired. Participants who remained cognitively unimpaired in comparison with those who progressed to CDR >0 were on average younger (73.1 vs. 75.4, *p* = 0.017), were more likely to be White (85.9% vs. 68.5%, *p* = 0.012), and had a lower frequency of APOE4 positivity (25.4% vs. 40.7%, *p* = 0.046). They also had higher FR scores (33.9 vs. 30.7, *p* < 0.001), but not TR scores (47.7 vs. 47.4, *p* = 0.050), than the participants who progressed. Additionally, participants who remained cognitively unimpaired had a higher proportion of individuals at lower SOMI stages (*p* = 0.004).

Raincloud plots for baseline neuroimaging biomarkers stratified by SOMI stage and by progression status (change in CDR) can be found in Supplementary Figures 1 and 2, respectively.

### SOMI predicts incident cognitive impairment

**Figure 2.**
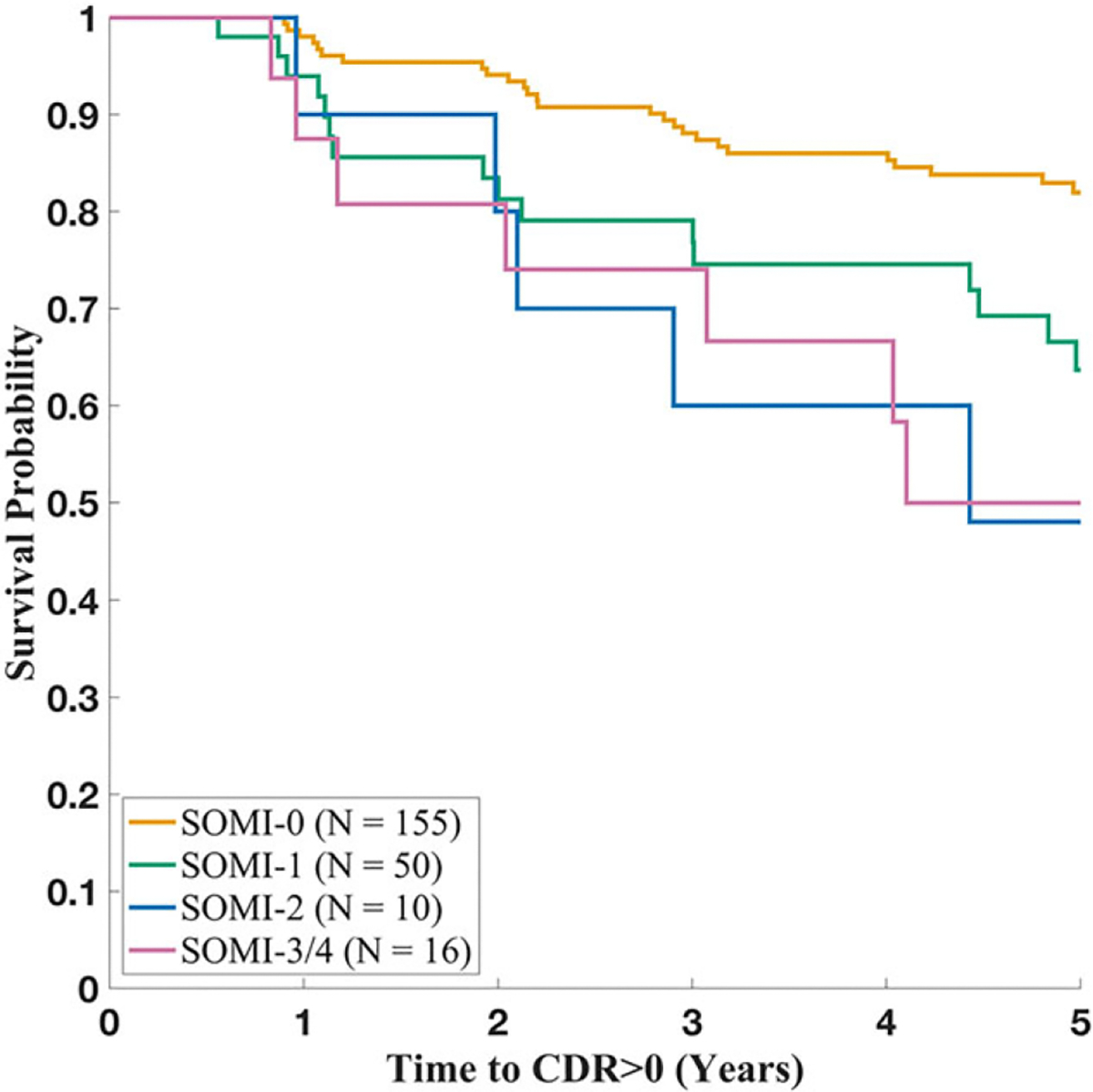
Kaplan-Meier survival curves stratified by SOMI stage at baseline.

Figure 2 illustrates Kaplan-Meier curves representing the association between SOMI stages and time to incident CDR >0 for the 231 participants. As depicted in this figure, cognitive impairment free survival declined least in the early stages of SOMI-0 and SOMI-1.

The Cox proportional hazards models, with Benjamini-Hochberg correction for multiple testing across 48 statistical tests, demonstrated robust associations between SOMI stages and incident cognitive impairment. Using SOMI-0 as the reference, the core model (Model 1) including age and APOE4 status found that SOMI-3/4 (HR = 3.73, CI = 1.58 – 8.79, *p* = 0.016 after correction) was significantly associated with incident cognitive impairment after multiple testing correction. SOMI-1 (HR = 2.06, CI = 1.09 – 3.88) and SOMI-2 (HR = 2.85, CI = 1.08 – 7.54) showed marginal associations (*p* = 0.059 and *p* = 0.065, respectively) after correction. Model 2 added PiB-PET global SUVR to the core model. SOMI-3/4 remained significant after correction (HR = 3.79, CI = 1.61 – 8.91, *p* = 0.015), while PiB-PET was not significant (HR = 1.21, CI = 0.94 – 1.57, *p* = 0.181 after correction). SOMI-1 and SOMI-2 remained marginally significant (*p* = 0.063 for both) after correction.

Model 3 included adjusted hippocampal volume. SOMI-1 (HR = 2.27, CI = 1.19 – 4.33, *p* = 0.051 after correction) and SOMI-3/4 (HR = 3.48, CI = 1.49 – 8.17, *p* = 0.022 after correction) showed marginal to significant associations after multiple testing correction. Hippocampal volume was marginally significant (HR = 0.76, CI = 0.57 – 1.02, *p* = 0.104 after correction).

Model 4 included entorhinal cortex thickness. SOMI-1 (HR = 2.52, CI = 1.32 – 4.81, *p* = 0.023 after correction) and SOMI-3/4 (HR = 5.61, CI = 2.33 – 13.51, *p* = 0.002 after correction) remained significant after multiple testing correction. Entorhinal thickness was highly significant (HR = 0.57, CI = 0.44 – 0.74, *p* = 0.001 after correction), representing the strongest biomarker association observed.

Model 5 incorporated FDG-PET measures. In this model, SOMI-3/4 (HR = 3.44, CI = 1.44 – 8.17, *p* = 0.022 after correction) remained significantly associated with incident cognitive impairment after multiple testing correction, while SOMI-1 (HR = 1.95, CI = 1.03 – 3.68, *p* = 0.065 after correction) and SOMI-2 (HR = 2.87, CI = 1.06 – 7.79, *p* = 0.065 after correction) showed a marginal association. Among the FDG-PET biomarkers, entorhinal FDG-PET showed a trend towards significance (HR = 0.62, CI = 0.42 – 0.92, *p* = 0.055 after correction). Other FDG-PET regions did not reach significance after correction.

Model 6 combined biomarkers demonstrated similar patterns with SOMI-3/4 (HR = 5.40, CI = 2.23 – 13.09, *p* = 0.002 after correction) and entorhinal thickness (HR = 0.63, CI = 0.49 – 0.80, *p* = 0.002 after correction) maintaining significance after multiple testing correction. Model 7 (best combination) showed SOMI-3/4 (HR = 5.30, CI = 2.16 – 13.00, *p* = 0.003 after correction) and entorhinal thickness (HR = 0.65, CI = 0.49 – 0.85, *p* = 0.015 after correction) remained significant.

Model performance was assessed using Maddala-Magee R^2^ indices. Model performance metrics across all analyses are summarized in Supplementary Table 1. The core model explained 8.93% of the variance, which increased to 9.75% with PiB-PET, 10.24% with hippocampal volume, 15.02% with entorhinal thickness, 11.19% with FDG-PET, 14.75% with entorhinal biomarkers, and 14.86% for Model 7. Partial likelihood ratio tests demonstrated significant model improvement, adding hippocampal volume (χ^2^ = 3.349, *p* = 0.067) showed a trend, while adding PiB-PET did not significantly improve the model (χ^2^ = 2.09, *p* = 0.148)

### Comprehensive model validation

To validate the robustness of SOMI’s predictive utility and address concerns about multiple testing and model overfitting, we conducted a comprehensive validation analysis across all seven models with systematic biomarker combinations. This analysis applied Benjamini-Hochberg correction for multiple testing across 48 statistical tests to ensure statistical rigor and minimize false discovery rates (Supplementary Table 2).

The validation demonstrated consistent patterns across models, with SOMI-3/4 maintaining significance after multiple testing correction in Models 1, 2, 3, 4, 6, and 7 (*p*-values ranging from 0.002 to 0.022 after correction). SOMI-1 achieved significance in Model 4 (*p* = 0.023 after correction) when entorhinal thickness was included, highlighting the enhanced discriminative power when combining cognitive staging with neuroimaging biomarkers.

Entorhinal cortex thickness emerged as the most robust biomarker predictor, maintaining significance across Models 4, 6, and 7 (*p*-values of 0.001, 0.002, and 0.015, respectively, after correction). This biomarker demonstrated hazard ratios consistently around 0.57 – 0.65, indicating that each standard deviation decrease in entorhinal thickness was associated with approximately 35 – 43% increased risk of cognitive impairment.

Model discrimination validation showed substantial improvement with biomarker integration. The Maddala-Magee R^2^ values demonstrated a clear hierarchy: core demographic model (8.93%), PiB-PET addition (9.75%), hippocampal volume (10.24%), entorhinal thickness (15.02%), FDG-PET (11.19%), combined biomarkers (14.75%), and best combination (14.86%). This pattern validates that cortical thickness measures, particularly entorhinal cortex, capture the most predictive neurobiological information for cognitive decline.

The validation confirmed that 11 of 48 statistical tests remained significant after Benjamini-Hochberg correction, with SOMI-3/4 and entorhinal thickness accounting for the majority of robust associations. This stringent multiple testing approach validates that the observed associations represent genuine predictive relationships rather than chance findings, supporting SOMI’s clinical utility for identifying individuals at elevated risk for cognitive decline despite the relatively low events-per-variable ratios in our models.

### Sensitivity analysis of defined outcome

To evaluate the robustness of findings using an alternative cognitive outcome, we conducted sensitivity analyses using CDR-SB ≥ 1 as the endpoint, which identified 37 events (16.0% event rate) among the 231 participants. After Benjamini-Hochberg correction across 48 statistical tests, fewer associations reached statistical significance compared to the primary CDR Global > 0 analysis. Complete results for the CDR-SB analysis are presented in Supplementary Table 3.

Entorhinal thickness emerged as the most robust predictor, maintaining significance in Model 4 (HR = 0.51, CI = 0.38 – 0.69, *p* < 0.001 after correction), Model 6 (HR = 0.59, CI = 0.44–0.79, *p* = 0.009 after correction), and Model 7 (HR = 0.60, CI = 0.44 – 0.83, *p* = 0.024 after correction). SOMI stages showed attenuated associations, with SOMI-3/4 demonstrating the strongest effect in Model 4 (HR = 5.31, CI = 1.88 – 15.02, *p* = 0.024 after correction), Model 6 (HR = 4.57, CI = 1.62–12.90, *p* = 0.033 after correction), and Model 7 (HR = 4.80, CI = 1.67 – 13.79, *p* = 0.033 after correction). SOMI-1 showed marginal associations in Model 4 (HR = 2.70, CI = 1.25 – 5.85, *p* = 0.075 after correction) but did not reach significance after multiple testing correction in other models.

Model discrimination was reduced compared to the primary analysis, with Maddala-Magee R^2^ values ranging from 0.047 for the core model to 0.086 for Model 4. The lower event rate (16.0% vs 23.4%) and more stringent outcome definition contributed to reduced statistical power in this sensitivity analysis. Despite attenuated statistical significance for SOMI stages, the consistency of hazard ratio point estimates and preservation of the risk gradient across SOMI categories supports the robustness of SOMI’s predictive relationship with cognitive decline across different operationalizations of cognitive impairment. Notably, entorhinal thickness maintained its strong predictive validity across all models where included, demonstrating the robustness of this biomarker across different cognitive outcome definitions.

### Sensitivity analysis with stricter enrollment criteria

To evaluate the robustness of SOMI’s predictive validity under more stringent cognitive screening and address concerns about baseline cognitive classification, we conducted sensitivity analyses excluding participants with moderate to severe memory impairment at baseline. Two screening thresholds were examined: participants performing within one standard deviation (SD =1) and two standard deviations (SD = 2) of age- and education-adjusted norms on all neuropsychological tests. Baseline characteristics for participants meeting stricter neuropsychological screening criteria are shown in Supplementary Table 4.

For stricter screening when SD = 1, this analysis included 110 participants with 16 events (14.5% event rate). Under these conditions, no participants were classified as SOMI-2 or SOMI-3/4 at baseline, leaving only SOMI-0 (*N* = 97) and SOMI-1 (*N* = 13) groups for analysis. After Benjamini-Hochberg correction across 48 statistical tests, no associations reached statistical significance. SOMI-1 showed hazard ratios ranging from 2.97 to 3.95 across models but did not achieve significance after multiple testing correction (*p*-values ranging from 0.096 to 0.121 after correction). Entorhinal thickness remained a predictor across Models 4, 6, and 7 with hazard ratios of 0.57, 0.60, and 0.56, respectively, but significance was attenuated after correction for multiple testing (*p* = 0.078 for all models after correction). Complete results for the SD =1 analysis are presented in Supplementary Table 5.

For screening when SD =2, this analysis included 193 participants with 39 events (20.2% event rate). The distribution included SOMI-0 (*N* = 137), SOMI-1 (*N* = 42), SOMI-2 (*N* = 6), and SOMI-3/4 (*N* = 8). After Benjamini-Hochberg correction across 48 statistical tests, SOMI-1 remained significantly associated with incident cognitive impairment across multiple models, with hazard ratios ranging from 2.89 to 3.77. SOMI-1 achieved significance in Models 3, 4, 6, and 7 (*p*-values of 0.027, 0.006, 0.013, and 0.013 respectively after correction). Entorhinal thickness demonstrated robust associations across models, with hazard ratios of 0.48, 0.55, and 0.57 and maintained significance after multiple testing correction in Models 4, 6, and 7 (*p*-values of 0.0002, 0.002, and 0.006 respectively after correction).

Model discrimination improved substantially under stricter screening criteria, with Maddala-Magee R^2^ values reaching 0.146 for the best combination model in the SD = 1 analysis and 0.160 for the thickness model in the SD = 2 analysis. The enhanced predictive validity under stricter screening criteria, particularly in the SD = 2 analysis where 7 of 48 tests remained significant after correction, supports that SOMI identifies genuine early markers of future decline rather than simply detecting pre-existing impairment misclassified as normal cognition.

Partial likelihood ratio tests showed that adding PiB-PET did not significantly improve model fit in either analysis (SD = 1: χ^2^ = 0.973, *p* = 0.324; SD = 2: χ^2^ = 1.014, *p* = 0.314), while adding hippocampal volume showed a trend toward significance in the SD = 2 analysis (χ^2^ = 3.428, *p* = 0.064). The complete results for both sensitivity analyses are presented in Supplementary Table 6.

### Incremental predictive value of SOMI

To evaluate SOMI’s incremental contribution to cognitive decline prediction beyond standard demographic and biomarker predictors, we conducted additional sensitivity analyses examining models without SOMI stages and without APOE4 status (Supplementary Tables 7 and 8).

Models excluding SOMI stages but including all biomarkers demonstrated substantially reduced predictive performance across all model configurations. Without SOMI, the core demographic model achieved a Maddala-Magee R^2^ of only 0.042 compared to 0.089 when SOMI was included (58% improvement). This pattern persisted across biomarker models: PiB-PET model (0.050 vs 0.098, 96% improvement), hippocampal volume model (0.054 vs 0.083, 54% improvement), thickness model (0.085 vs 0.136, 60% improvement), FDG-PET model (0.070 vs 0.093, 33% improvement), combined biomarkers model (0.087 vs 0.132, 52% improvement), and best combination model (0.090 vs 0.138, 53% improvement).

Models excluding APOE4 status showed more modest reductions in predictive performance, with R^2^ values decreasing by approximately 10–15% across models. Notably, entorhinal thickness remained the strongest biomarker predictor even without APOE4 (HR = 0.65, CI = 0.50 – 0.83), while PiB-PET showed enhanced significance when APOE4 was excluded (HR = 1.17, CI = 0.95 – 1.44), consistent with known shared variance between APOE4 and amyloid burden.

## Discussion

The results show that the SOMI system identifies a subset of individuals considered cognitively normal by conventional neuropsychological tests including delayed story recall and CDR scale who have subtle memory impairment and are at risk of incident cognitive impairment. After Benjamini-Hochberg correction for multiple testing, the HRs for progression to CDR >0 within 5 years using SOMI-0 as a reference were 2.06, 2.85, and 3.73 for SOMI-1, SOMI-2, and SOMI-3/4, respectively, with SOMI-3/4 achieving statistical significance.

The robustness of these findings was enhanced by our implementation of Benjamini-Hochberg correction for multiple testing across 48 statistical tests, controlling the false discovery rate at 5%. This conservative statistical approach addresses concerns about Type I error inflation that can arise from testing multiple models and biomarker combinations. Despite this stringent correction, SOMI-3/4 maintained significance across most models, and entorhinal thickness emerged as the most robust biomarker predictor, demonstrating the genuine nature of these associations rather than chance findings. The systematic validation across seven different model configurations provides strong evidence for SOMI’s clinical utility even under rigorous statistical scrutiny.

Adding all the biomarkers including PiB-PET, adjusted hippocampal volume, cortical thickness measures, and FDG-PET to the base model nearly doubled the explained variance. Importantly, the HRs associated with SOMI stages generally remained consistent or even increased, indicating that SOMI enhances prediction beyond biomarkers. Among the imaging biomarkers we considered, entorhinal thickness emerged as the most robust predictor, maintaining significance across multiple models after Benjamini-Hochberg correction (HR = 0.57 – 0.65, *p* ≤ 0.015), while entorhinal FDG-PET showed significance in specific models. The same measures for the inferior temporal region, hippocampal volume and PiB-PET were not significant predictors. Our findings were strengthened by comprehensive multiple testing correction and sensitivity analyses that confirmed the robustness of key associations.

The systematic evaluation of model performance using Maddala-Magee R^2^ indices revealed important insights into the relative contributions of different biomarker modalities. While the core model explained 8.93% of variance, the addition of entorhinal thickness nearly doubled this to 15.02%, representing the largest single improvement among all biomarkers tested. This substantial enhancement in predictive performance underscores the critical importance of entorhinal cortex integrity in early cognitive decline prediction (Xie et al., 2025). Notably, the combination of multiple biomarkers (14.86% variance explained) did not substantially improve upon entorhinal thickness alone, suggesting that this single measure captures much of the predictive neurobiological information relevant to cognitive decline risk.

The current longitudinal findings complement our previous cross-sectional work by demonstrating that SOMI’s associations with neuroimaging biomarkers translate into meaningful prediction of future cognitive decline, with entorhinal thickness serving as both a cross-sectional correlate and longitudinal predictor. In a subset of the current HABS cohort, we found that participants with memory storage impairments (SOMI-3/4) had higher entorhinal and inferior temporal tau burden as well as smaller hippocampal volumes than those without memory impairment (SOMI-0) or subtle retrieval impairment (SOMI-1) (Grober et al., 2021). The current study extends these cross-sectional associations by showing that entorhinal cortex thickness not only differentiates SOMI stages at baseline but also independently predicts cognitive decline over time, with hazard ratios of 0.57 – 0.65 across models after multiple testing correction. This convergence of cross-sectional biomarker associations and longitudinal predictive validity strengthens the evidence for SOMI’s clinical utility.

This is the second demonstration that cognitively unimpaired individuals in higher SOMI stages have an elevated risk of developing cognitive impairment. In the Knight ADRC cohort where 969 participants were followed for 10 years, the risk of progression to CDR >0 doubled for those in SOMI-2 and tripled for those in SOMI-3/4 with and without the inclusion of CSF measures of *β*-amyloid and tau pathology or hippocampal volume (Grober et al., 2023). Using data from several longitudinal cohorts, we have previously shown that some participants presumed to be cognitively unimpaired at baseline based on conventional criteria, had moderate memory retrieval impairment (SOMI-2) and/or mild storage impairment (SOMI-3,4). They comprised 20% of the A4 cohort, 16% of the Baltimore Longitudinal Study of Aging (BLSA), and 18% Knight ADRC (Grober et al., 2022, Petersen et al., 2023). The prevalence of such participants is consistent with the growing documentation of cognitive deficits in ostensibly cognitively unimpaired participants.

The comprehensive sensitivity analyses conducted across multiple outcome definitions and screening criteria provide strong evidence for SOMI’s robustness and clinical validity. When examining CDR-SB ≥1 as an alternative outcome, SOMI stages maintained consistent directional effects despite reduced statistical power from lower event rates (16.0% vs 23.4%), confirming that the predictive relationship persists across different operationalizations of cognitive decline. More importantly, the stricter baseline screening analyses revealed that SOMI’s predictive validity actually strengthened when participants with moderate to severe memory impairment were excluded.

Under SD = 2 screening criteria, SOMI-1 achieved significance across multiple models with hazard ratios ranging from 2.89 to 3.77, and model discrimination improved substantially (Maddala-Magee R^2^ reaching 0.160). This enhanced performance under stricter screening provides compelling evidence that SOMI identifies genuine early markers of future decline rather than simply detecting pre-existing impairment misclassified as normal cognition.

The incremental predictive value analyses demonstrated SOMI’s substantial independent contribution beyond standard demographic and biomarker predictors. Models excluding SOMI stages showed dramatically reduced predictive performance, with improvements ranging from 33 to 96% when SOMI was included across different biomarker configurations. This finding is particularly clinically relevant, as it suggests that cognitive staging through SOMI provides unique predictive information that cannot be captured by neuroimaging biomarkers alone. The relatively modest impact of excluding APOE4 status (10 – 15% reduction in R^2^) compared to excluding SOMI stages highlights the superior predictive utility of cognitive performance measures over genetic risk factors in this context.

Lack of significance in association of cross-sectional hippocampal volume with longitudinal cognitive outcomes in this study is not entirely surprising and is consistent with our previous findings in a different population-based study (Petersen et al., 2023). Hippocampal volume has some limitations as a cross-sectional marker of neurodegeneration as hippocampal atrophy may be due to diseases other than AD (Aschenbrenner et al., 2018). Lastly, similar to previous findings, we found APOE4 positivity to only be associated with incident cognitive impairment in the absence of PiB-PET (Grober et al., 2023). This finding is reasonably explained by shared variance between APOE4 and biomarkers for amyloid (Hong et al., 2022) as well as the known interaction between APOE4 positivity and amyloid in association with memory and cognition (Lim et al., 2016, Mormino et al., 2014b).

In addition to in vivo AD biomarkers, SOMI is also associated with the likelihood of AD neuropathology in the Washington University autopsy series (Grober et al, 2021). Cases with subtle (SOMI-3) and moderately impaired storage (SOMI-4) were 4 times as likely to have moderate or high levels of AD neuropathologic change than cases with no memory impairment (SOMI-0). Those with moderate storage impairment (SOMI-4) were nearly six times as likely to have more advanced Braak neurofibrillary tau pathology.

This study has limitations. First, we selected regions of interest (ROIs) that have been demonstrated to be associated with cognitive decline. Including additional ROIs from MRI and FDG-PET may provide additional insight. Second, the number of participants that were in the higher SOMI stages was small, limiting our ability to perform additional stratified analyses. Third, the small number of participants with RISU profiles (*n* = 29) precluded analysis of this potentially important subgroup, which warrants further study. Fourth, the study is limited by relatively low EPV ratios, particularly for complex models, which may affect the stability of hazard ratio estimates. However, we implemented multiple testing correction and sensitivity analyses to support our primary findings. Lastly, the HABS cohort largely consists of highly educated participants who were White and healthy. Additional analysis in more diverse samples is needed to generalize these findings to clinical settings. The structured encoding and semantic cueing of the FCSRT may reduce cultural and educational biases compared to traditional memory tests. Additionally, our previous work in the Knight ADRC cohort, which included greater demographic diversity than HABS, demonstrated similar SOMI stage distributions and comparable predictive validity for cognitive decline (Grober et al., 2023).

In conclusion, we have demonstrated that episodic memory impairment defined by SOMI and entorhinal cortex thickness independently and robustly predict incident cognitive impairment among individuals presumed to be cognitively normal. The prognostic value of SOMI, validated through comprehensive sensitivity analyses and multiple testing correction, combined with its associations with in vivo imaging and postmortem AD neuropathology, supports its implementation in secondary prevention trials and clinical practice. The predictive value of SOMI beyond standard demographic and biomarker predictors underscores its potential as a cost-effective screening tool for identifying individuals at risk for cognitive decline.

## Supplementary Material

Supplement

**Supplementary material.** The supplementary material for this article can be found at https://doi.org/10.1017/S1355617725101240.

## Figures and Tables

**Table 1.** Stages of objective memory impairment. SOMI stages defined by free recall and total recall score ranges and years to diagnosis on the picture version of the free and cued selective reminding test with immediate recall (pFCSRT+IR).

	Stages of objective memory impairment (SOMI)	Free recall scores	Total recall scores	Class of memory impairment

0	No memory impairment	>30	>46	None detected by pFCSRT+IR
1	Subtle retrieval impairment	25 – 30	>46	Free recall declines at a constant rate. Storage is preserved.
2	Moderate retrieval impairment	20–24	>46	Rate of free recall decline doubles. Executive dysfunction accelerates. Storage is preserved.
3	Subtle storage impairment	any	45–46	Cuing fails to normalize total recall.
4	Significant storage impairment compatible with Dementia	any	33–44	Cognitive decline accelerates heralding ADL impairment.

*Notes:* A subset of participants does not meet the SOMI criteria as summarized in the table (FR <20 and TR >46). Retrieval is impaired but storage is unimpaired (RISU).

**Table 2.** Characteristics table for the participants

		Entire sample	Remains CDR = 0	Changes to CDR >0	*p*

*N*, (%)		231	177 (76.6%)	54 (23.4%)	
Age, years, mean (SD)		73.7 (6.0)	73.1 (5.8)	75.4 (6.3)	0.017
Female, *N*(%)		139 (60.2%)	107 (60.5%)	32 (59.3%)	0.999
Education, years, mean (SD)		15.9 (2.9)	16.0 (3.0)	15.5 (2.8)	0.312
White, *N*(%)		189 (81.8%)	152 (85.9%)	37 (68.5%)	0.012
Black, *N*(%)		35 (15.2%)	22 (12.4%)	13 (24.1%)	
APOE4+, *N*(%)		67 (29.0%)	45 (25.4%)	22 (40.7%)	0.046
FR, mean (SD)		33.1 (5.4)	33.9 (4.8)	30.7 (6.3)	<0.001
TR, mean (SD)		47.7 (0.9)	47.7 (0.8)	47.4 (1.2)	0.050
Digit symbol substition (DSS), mean (SD)		47.2 (10.7)	47.8 (11.0)	45.5 (9.3)	0.184
Trail making test B (TMT-B), mean (SD)		92.8 (50.1)	89.4 (46.9)	103.7 (58.0)	0.066
EF/PS composite, mean (SD)		0.00 (0.85)	0.06 (0.85)	−0.19 (0.85)	0.063
SOMI-0, *N*(%)		155	129 (83.2%)	26 (16.8%)	0.004
SOMI-1, *N*(%)		50	34 (68.0%)	16 (32.0%)	
SOMI-2, *N*(%)		10	5 (50.0%)	5 (50.0%)	
SOMI-3/4, *N*(%)		16	9 (56.3%)	7 (43.8%)	
PiB DVR, mean (SD)		1.17 (0.19)	1.15 (0.18)	1.24 (0.21)	0.003
MRI	HVa, cm^3^, mean (SD)	7.42 (0.69)	7.49 (0.67)	7.20 (0.71)	0.008
	Entorhinal thickness, mm, mean (SD)	6.76 (0.65)	6.85 (0.60)	6.47 (0.72)	<0.001
	Inf. temporal thickness, mm, mean (SD)	5.45 (0.26)	5.45 (0.24)	5.44 (0.33)	0.709
FDG	Hippocampus, FDG SUVr, mean (SD)	0.85 (0.06)	0.85 (0.05)	0.85 (0.07)	0.999
	Entorhinal, FDG SUVr, mean (SD)	0.73 (0.07)	0.74 (0.06)	0.71 (0.09)	0.035
	Inf. temporal, FDG SUVr, mean (SD)	1.00 (0.08)	1.00 (0.08)	0.99 (0.07)	0.386

**Table 3.** Hazard ratios from the Cox proportional hazard models

Variable	1 Core	2 +PIB	3 +HV	4 +Thickness	5 +FDG	6 +Entorhinal	7 +PIB + HV +Entorhinal

Age	1.04 (1.00 – 1.09)	1.04 (0.99 – 1.09)	1.02 (0.97 – 1.08)	1.04 (0.99 – 1.09)	1.04 (0.99 – 1.09)	1.03 (0.98 – 1.08)	1.03 (0.98 – 1.08)
APOE4+	1.90 (1.09 – 3.32)†	1.49 (0.77 – 2.87)	1.90 (1.09 – 3.31)†	1.77 (1.01 – 3.10)†	1.92 (1.09 – 3.39)†	1.82 (1.04 – 3.19)†	1.71 (0.92 – 3.17)
SOMI-1	2.06 (1.09 – 3.88)†	2.00 (1.07 – 3.77)†	2.27 (1.19 – 4.33)†	2.52 (1.32 – 4.81)*	1.95 (1.03 – 3.68)†	2.24 (1.18 – 4.27)†	2.21 (1.16 – 4.22)†
SOMI-2	2.85 (1.08 – 7.54)†	2.90 (1.10 – 7.66)†	2.79 (1.06 – 7.35)†	2.97 (1.11 – 7.93)†	2.87 (1.06 – 7.79)†	3.13 (1.17 – 8.39)†	3.14 (1.17 – 8.40)†
SOMI-3/4	3.73 (1.58 – 8.79)*	3.79 (1.61 – 8.91)*	3.48 (1.49 – 8.17)*	5.61 (2.33 – 13.51)**	3.44 (1.44 – 8.17)*	5.40 (2.23 – 13.09)**	5.30 (2.16 – 13.00)**
PIB global		1.21 (0.94 – 1.57)					1.06 (0.82 – 1.38)
Hippocampal volume			0.76 (0.57 – 1.02)				0.97 (0.71 – 1.33)
Entorhinal thickness				0.57 (0.44 – 0.74)**		0.63 (0.49 – 0.80)**	0.65 (0.49 – 0.85)*
Inferior temporal thickness				1.23 (0.93 – 1.63)			
Entorhinal FDG					0.62 (0.42 – 0.92)†	0.85 (0.64 – 1.12)	0.85 (0.64 – 1.14)
Inferior temporal FDG					1.03 (0.73 – 1.45)		
Hippocampal FDG					1.37 (0.91 – 2.07)		

*Note:* Model 1 only includes SOMI stage, age, and APOE4 status. Models 2 and 3 also include PIB and adjusted hippocampal volume (HVa), respectively. Models 4 and 5 include, in addition to the core model, either thickness or FDG measures, respectively. Model 6 includes the core model with all entorhinal measures. Model 7 includes the core model, PIB, HVa, and entorhinal measures. †, *, **, and *** indicate *p*-values less than 0.10, 0.05, 0.01, and 0.001, respectively, after Benjamini-Hochberg correction for multiple testing across 48 statistical tests.

## Data Availability

The data used for the purpose of this study is available from the HABS website (habs.mgh.harvard.edu) for eligible researchers through an online data request process. We used the Strengthening the Reporting of Observational Studies in Epidemiology cohort checklist when writing our report (von Elm et al., 2007). The HABS research was completed in accordance with Helsinki Declaration.

## Statement of Research Significance

### Research Question:

We investigated the Stages of Objective Memory Impairment (SOMI) system prediction of incident cognitive impairment (CDR >0) in combination with demographic features and neuroimaging biomarkers.

### Main Findings:

After multiple testing corrections, the HRs for progression to CDR >0 within 5 years using SOMI-0 as a reference were 2.06, 2.85, and 3.73 for SOMI-1, SOMI-2, and SOMI-3/4, respectively, with SOMI-3/4 achieving statistical significance across most biomarker models. Entorhinal thickness emerged as the most robust biomarker predictor (HR = 0.57 – 0.65, *p* ≤ 0.015).
