# Supplementary material for: Assessing Stages of Objective Memory Impairment and neuroimaging as risk factors of incident cognitive impairment
Source: J Int Neuropsychol Soc. Author manuscript; Available in PMC 2025 Sep 10. (PMC12418808; doi:10.1017/S1355617725101240)
Supplement: Supplement [file NIHMS2106822-supplement-Supplement.docx]

**Supplementary Figure 1. Raincloud plots for each neuroimaging biomarker at baseline stratified by SOMI stage.**

**
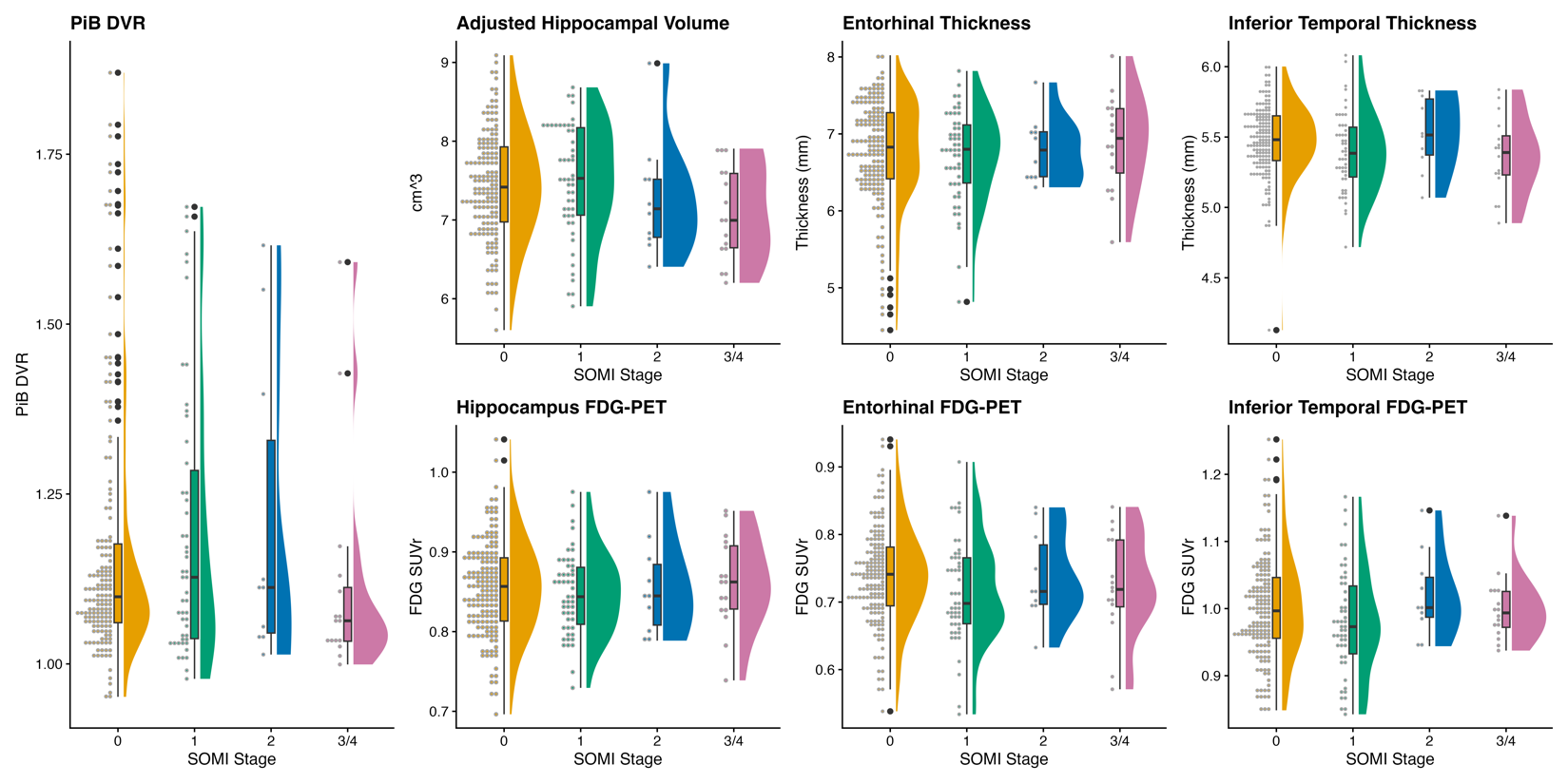
**

**Supplementary Figure 2. Raincloud plots for each neuroimaging biomarker at baseline stratified by people that remains at CDR=0 and progress to CDR>0.**

**
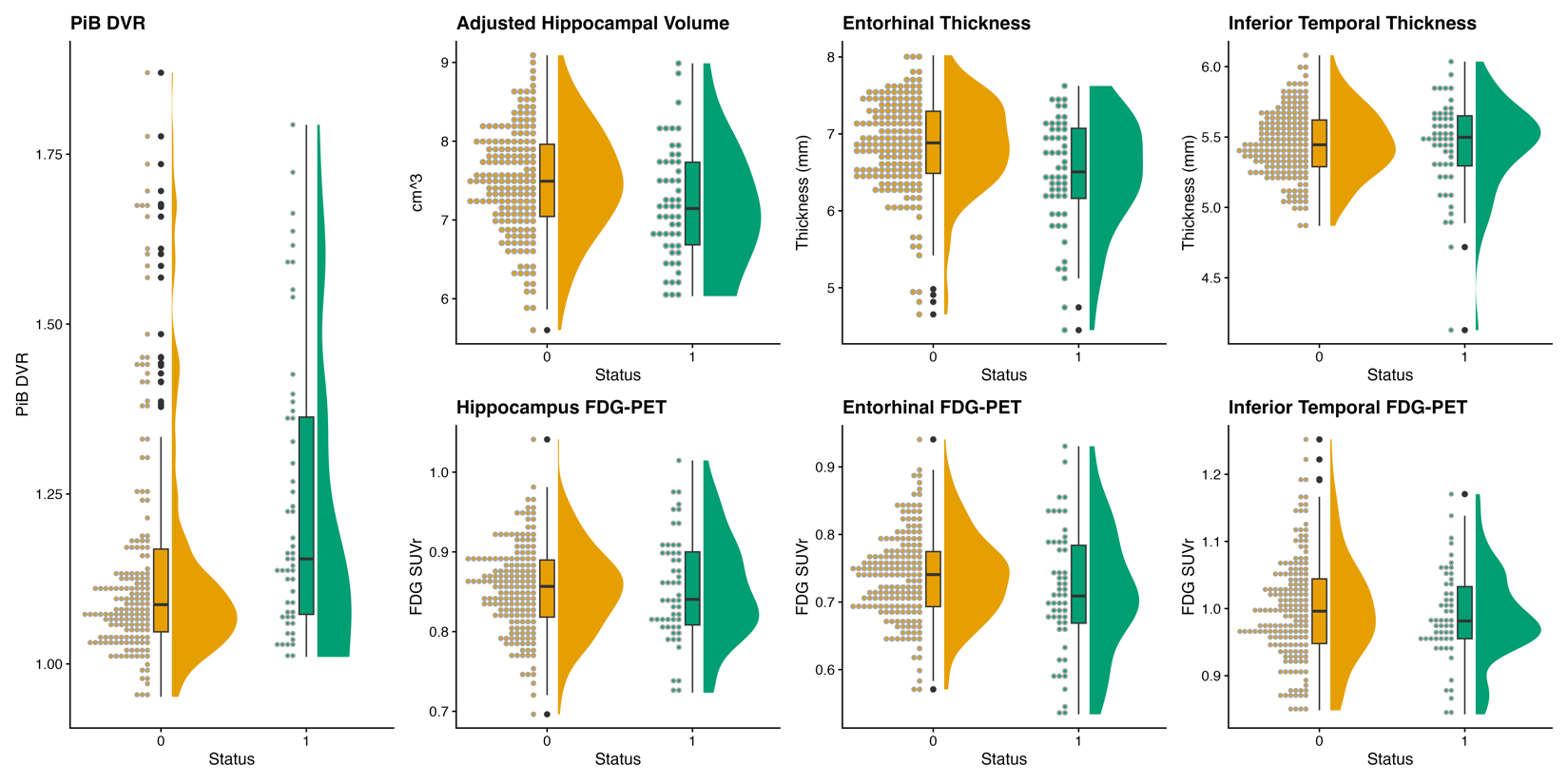
**

**Supplemental Table 1. Model performance metrics comparison across analyses.** Maddala-Magee R² values (expressed as percentages) for each Cox proportional hazards model across primary and sensitivity analyses.

| **Model** | **Primary Analysis (CDR>0)** | **CDR-SB Sensitivity** | **Stricter Screening (SD=1)** | **Stricter Screening (SD=2)** |
| --- | --- | --- | --- | --- |
| **Model 1: Core** | 8.90% | 3.70% | 9.20% | 7.10% |
| **Model 2: +PIB** | 9.80% | 5.20% | 10.00% | 7.60% |
| **Model 3: +HV** | 10.20% | 4.10% | 9.70% | 8.70% |
| **Model 4: +Thickness** | 15.00% | 11.20% | 14.40% | 16.00% |
| **Model 5: +FDG** | 11.20% | 4.90% | 12.80% | 8.30% |
| **Model 6: +Entorhinal** | 14.80% | 8.80% | 14.10% | 14.40% |
| **Model 7: +PIB+HV+Entorhinal** | 14.90% | 9.20% | 14.60% | 14.70% |

**Supplemental Table 2.** **Multiple testing correction summary across all analyses**. Number of statistical tests and significant associations before and after Benjamini-Hochberg correction for false discovery rate control at α = 0.05 across primary and sensitivity analyses.

| **Analysis** | **Total Tests** | **Significant Before Correction** | **Significant After Correction** | **% Surviving Correction** | **α Level** |
| --- | --- | --- | --- | --- | --- |
| **Primary Analysis (CDR>0)** | 48 | 30 | 11 | 36.70% | 0.05 |
| **CDR-SB Sensitivity** | 48 | 14 | 6 | 42.90% | 0.05 |
| **Stricter Screening (SD=1)** | 48 | 13 | 0 | 0.00% | 0.05 |
| **Stricter Screening (SD=2)** | 48 | 12 | 7 | 58.30% | 0.05 |

**Supplemental Table 3. CDR-SB outcome analysis.** Hazard ratios and 95% confidence intervals from Cox proportional hazards models predicting incident cognitive impairment defined as CDR Sum-of-Boxes ≥ 1.

| Variable | **1**  **Core** | **2**  **+PIB** | **3**  **+HV** | **4**  **+Thickness** | **5**  **+FDG** | **6**  **+Entorhinal** | **7**  **+PIB+HV**  **+Entorhinal** |
| --- | --- | --- | --- | --- | --- | --- | --- |
| Age | 1.02 (0.97-1.08) | 1.02 (0.96-1.07) | 1.01 (0.95-1.07) | 1.02 (0.97-1.07) | 1.02 (0.96-1.08) | 1.00 (0.95-1.06) | 1.01 (0.95-1.07) |
| APOE4+ | 1.09 (0.53-2.23) | 0.72 (0.31-1.68) | 1.10 (0.54-2.25) | 0.92 (0.45-1.91) | 1.09 (0.53-2.27) | 0.97 (0.47-2.00) | 0.82 (0.37-1.80) |
| SOMI-1 | 2.04 (0.94-4.41) | 1.97 (0.91-4.24) | 2.16 (0.99-4.73) | 2.70 (1.25-5.85)† | 2.03 (0.94-4.40) | 2.33 (1.07-5.07) | 2.18 (1.00-4.75) |
| SOMI-2 | 2.65 (0.77-9.14) | 2.70 (0.78-9.35) | 2.55 (0.74-8.81) | 2.90 (0.83-10.14) | 2.66 (0.76-9.38) | 3.38 (0.95-11.94) | 3.39 (0.95-12.07) |
| SOMI-3/4 | 3.14 (1.13-8.69) | 3.30 (1.19-9.10) | 3.00 (1.09-8.27) | 5.31 (1.88-15.02)* | 2.93 (1.05-8.20) | 4.57 (1.62-12.90)* | 4.80 (1.67-13.79)* |
| PIB Global |  | 1.38 (1.01-1.89) |  |  |  |  | 1.19 (0.87-1.64) |
| Hippocampal Volume |  |  | 0.83 (0.58-1.19) |  |  |  | 1.10 (0.75-1.60) |
| Entorhinal Thickness |  |  |  | 0.51 (0.38-0.69)*** |  | 0.59 (0.44-0.79)** | 0.60 (0.44-0.83)* |
| Inferior Temporal Thickness |  |  |  | 1.54 (1.10-2.15)† |  |  |  |
| Entorhinal FDG |  |  |  |  | 0.66 (0.41-1.07) | 0.94 (0.67-1.32) | 0.96 (0.68-1.36) |
| Inferior Temporal FDG |  |  |  |  | 1.12 (0.74-1.69) |  |  |
| Hippocampal FDG |  |  |  |  | 1.30 (0.80-2.11) |  |  |

Note: Model 1 only includes SOMI stage, age, and APOE4 status. Models 2 and 3 also include PIB and adjusted hippocampal volume (HVa), respectively. Models 4 and 5 include, in addition to the core model, either thickness or FDG measures, respectively. Model 6 includes the core model with all entorhinal measures. Model 7 includes the core model, PIB, HVa, and entorhinal measures. †, *, **, and *** indicate p-values less than 0.10, 0.05, 0.01, and 0.001, respectively, after Benjamini-Hochberg correction for multiple testing across 48 statistical tests.

**Supplemental Table 4. Characteristics table for entire sample and stricter neuropsychological screening criteria.** Baseline demographics, cognitive performance, and biomarker measures for the entire sample (N=231) and subsets meeting stricter neuropsychological screening criteria requiring performance within 1 standard deviation (SD=1, N=110) and 2 standard deviations (SD=2, N=193) of age- and education-adjusted norms on all tests. Continuous variables presented as mean (SD); categorical variables as N (%). P-values from ANOVA for continuous variables and chi-square/Fisher's exact tests for categorical variables.

| **Variable** | **Entire Sample** | **SD=1 Screening** | **SD=2 Screening** | **p** |
| --- | --- | --- | --- | --- |
| **N, (%)** | 230 | 110 | 193 |  |
| **Age, years, mean (SD)** | 73.6 (6.0) | 73.0 (6.0) | 73.5 (6.0) | 0.697 |
| **Female, N(%)** | 138 (60.0%) | 69 (62.7%) | 116 (60.1%) | 0.877 |
| **Education, years, mean (SD)** | 15.9 (2.9) | 16.3 (2.6) | 15.9 (2.9) | 0.497 |
| **White, N(%)** | 189 (82.2%) | 98 (89.1%) | 168 (87.0%) | 0.171 |
| **Black, N(%)** | 35 (15.2%) | 10 (9.1%) | 20 (10.4%) | 0.169 |
| **APOE4+, N(%)** | 67 (29.1%) | 31 (28.2%) | 58 (30.1%) | 0.941 |
| **FR, mean (SD)** | 33.1 (5.4) | 35.7 (3.7) | 33.9 (4.8) | <0.001 |
| **TR, mean (SD)** | 47.7 (0.9) | 47.9 (0.3) | 47.8 (0.5) | 0.005 |
| **Digit Symbol Substitution (DSS), mean (SD)** | 47.3 (10.7) | 50.9 (9.8) | 48.6 (10.4) | 0.010 |
| **Trail Making Test B (TMT-B), mean (SD)** | 92.1 (49.2) | 71.3 (22.6) | 80.0 (30.2) | <0.001 |
| **SOMI-0** | 155 | 97 | 139 | <0.001 |
| **SOMI-1** | 49 | 13 | 42 | 0.073 |
| **SOMI-2** | 10 | 0 | 5 | 0.061 |
| **SOMI-3/4** | 16 | 0 | 7 | 0.006 |
| **PiB DVR, mean (SD)** | 1.2 (0.2) | 1.2 (0.2) | 1.2 (0.2) | 0.927 |
| **HVa, cm^3^, mean (SD)** | 7.4 (0.7) | 7.5 (0.7) | 7.5 (0.7) | 0.889 |
| **Entorhinal Thickness, mm, mean (SD)** | 6.8 (0.7) | 6.8 (0.7) | 6.8 (0.6) | 0.957 |
| **Inf. Temporal Thickness, mm, mean (SD)** | 5.5 (0.3) | 5.5 (0.3) | 5.5 (0.3) | 0.896 |
| **Hippocampus, FDG SUVR, mean (SD)** | 0.9 (0.1) | 0.9 (0.1) | 0.9 (0.1) | 0.770 |
| **Entorhinal, FDG SUVR, mean (SD)** | 0.7 (0.1) | 0.7 (0.1) | 0.7 (0.1) | 0.845 |
| **Inf. Temporal, FDG SUVR, mean (SD)** | 1.0 (0.1) | 1.0 (0.1) | 1.0 (0.1) | 0.919 |

**Supplemental Table 5. Stricter screening analysis (SD=1).** Hazard ratios and 95% confidence intervals from Cox proportional hazards models in participants meeting stricter neuropsychological screening criteria (performance within 1 standard deviation of age- and education-adjusted norms).

| Variable | **1**  **Core** | **2**  **+PIB** | **3**  **+HV** | **4**  **+Thickness** | **5**  **+FDG** | **6**  **+Entorhinal** | **7**  **+PIB+HV**  **+Entorhinal** |
| --- | --- | --- | --- | --- | --- | --- | --- |
| Age | 1.02 (0.94-1.11) | 1.01 (0.93-1.10) | 1.00 (0.91-1.11) | 1.02 (0.94-1.12) | 1.03 (0.94-1.12) | 1.01 (0.93-1.10) | 1.02 (0.93-1.12) |
| APOE4+ | 3.89 (1.42-10.67)† | 3.02 (0.96-9.44) | 3.91 (1.42-10.76)† | 3.05 (1.10-8.52)† | 3.94 (1.40-11.07)† | 3.17 (1.12-8.97)† | 2.85 (0.94-8.67) |
| SOMI-1 | 3.61 (1.10-11.80)† | 3.95 (1.18-13.15)† | 3.69 (1.12-12.19)† | 3.61 (1.13-11.55)† | 2.97 (0.78-11.21) | 3.67 (1.00-13.44) | 3.75 (1.00-13.98) |
| SOMI-2 |  |  |  |  |  |  |  |
| SOMI-3/4 |  |  |  |  |  |  |  |
| PIB Global |  | 1.27 (0.80-2.01) |  |  |  |  | 1.14 (0.71-1.84) |
| Hippocampal Volume |  |  | 0.83 (0.49-1.39) |  |  |  | 1.21 (0.66-2.24) |
| Entorhinal Thickness |  |  |  | 0.57 (0.37-0.86)† |  | 0.60 (0.39-0.91)† | 0.56 (0.33-0.95)† |
| Inferior Temporal Thickness |  |  |  | 1.16 (0.70-1.92) |  |  |  |
| Entorhinal FDG |  |  |  |  | 0.58 (0.32-1.08) | 0.99 (0.57-1.71) | 0.99 (0.57-1.71) |
| Inferior Temporal FDG |  |  |  |  | 0.94 (0.53-1.67) |  |  |
| Hippocampal FDG |  |  |  |  | 1.86 (0.87-3.97) |  |  |

Note: Model 1 only includes SOMI stage, age, and APOE4 status. Models 2 and 3 also include PIB and adjusted hippocampal volume (HVa), respectively. Models 4 and 5 include, in addition to the core model, either thickness or FDG measures, respectively. Model 6 includes the core model with all entorhinal measures. Model 7 includes the core model, PIB, HVa, and entorhinal measures. †, *, **, and *** indicate p-values less than 0.10, 0.05, 0.01, and 0.001, respectively, after Benjamini-Hochberg correction for multiple testing across 48 statistical tests.

**Supplemental Table 6. Stricter screening analysis (SD=2).** Hazard ratios and 95% confidence intervals from Cox proportional hazards models in participants meeting stricter neuropsychological screening criteria (performance within 2 standard deviations of age- and education-adjusted norms).

| Variable | **1**  **Core** | **2**  **+PIB** | **3**  **+HV** | **4**  **+Thickness** | **5**  **+FDG** | **6**  **+Entorhinal** | **7**  **+PIB+HV**  **+Entorhinal** |
| --- | --- | --- | --- | --- | --- | --- | --- |
| Age | 1.04 (0.99-1.10) | 1.04 (0.98-1.10) | 1.01 (0.95-1.08) | 1.04 (0.99-1.10) | 1.04 (0.98-1.10) | 1.02 (0.97-1.08) | 1.02 (0.96-1.08) |
| APOE4+ | 1.86 (0.97-3.57) | 1.54 (0.72-3.29) | 1.99 (1.04-3.82) | 1.73 (0.90-3.32) | 1.83 (0.95-3.54) | 1.76 (0.91-3.38) | 1.63 (0.79-3.37) |
| SOMI-1 | 2.42 (1.22-4.80)† | 2.41 (1.22-4.78)† | 2.89 (1.41-5.93)* | 3.77 (1.82-7.81)** | 2.31 (1.16-4.60)† | 3.30 (1.57-6.93)* | 3.46 (1.62-7.38)* |
| SOMI-2 | 2.22 (0.51-9.71) | 2.41 (0.55-10.57) | 2.39 (0.55-10.51) | 2.31 (0.52-10.18) | 2.18 (0.48-10.02) | 2.58 (0.58-11.46) | 2.81 (0.62-12.73) |
| SOMI-3/4 | 2.01 (0.45-9.05) | 2.08 (0.46-9.35) | 2.39 (0.53-10.74) | 4.02 (0.87-18.51) | 1.76 (0.39-7.96) | 3.67 (0.80-16.92) | 3.90 (0.84-18.16) |
| PIB Global |  | 1.17 (0.87-1.58) |  |  |  |  | 1.09 (0.80-1.48) |
| Hippocampal Volume |  |  | 0.71 (0.50-1.02) |  |  |  | 0.91 (0.62-1.33) |
| Entorhinal Thickness |  |  |  | 0.48 (0.35-0.65)*** |  | 0.55 (0.41-0.74)** | 0.57 (0.42-0.78)** |
| Inferior Temporal Thickness |  |  |  | 1.39 (1.01-1.93) |  |  |  |
| Entorhinal FDG |  |  |  |  | 0.69 (0.44-1.09) | 0.90 (0.66-1.24) | 0.93 (0.66-1.29) |
| Inferior Temporal FDG |  |  |  |  | 1.03 (0.68-1.56) |  |  |
| Hippocampal FDG |  |  |  |  | 1.22 (0.73-2.04) |  |  |

Note: Model 1 only includes SOMI stage, age, and APOE4 status. Models 2 and 3 also include PIB and adjusted hippocampal volume (HVa), respectively. Models 4 and 5 include, in addition to the core model, either thickness or FDG measures, respectively. Model 6 includes the core model with all entorhinal measures. Model 7 includes the core model, PIB, HVa, and entorhinal measures. †, *, **, and *** indicate p-values less than 0.10, 0.05, 0.01, and 0.001, respectively, after Benjamini-Hochberg correction for multiple testing across 48 statistical tests.

**Supplementary Table 7. Hazard ratios from the Cox Proportional Hazard Models without APOE4 status.**

| Variable | **1**  **Core** | **2**  **+PIB** | **3**  **+HV** | **4**  **+Thickness** | **5**  **+FDG** | **6**  **+Entorhinal** | **7**  **+PIB+HV**  **+Entorhinal** |
| --- | --- | --- | --- | --- | --- | --- | --- |
| Age | 1.04 (0.99-1.09) | 1.04 (0.99-1.09) | 1.02 (0.97-1.08) | 1.04 (0.99-1.09) | 1.03 (0.98-1.09) | 1.03 (0.98-1.08) | 1.03 (0.98-1.08) |
| APOE4+ |  |  |  |  |  |  |  |
| SOMI-1 | 1.97 (1.04-3.73)† | 1.96 (1.04-3.68)† | 2.13 (1.11-4.07)* | 2.36 (1.24-4.50)* | 1.92 (1.01-3.65)† | 2.13 (1.12-4.03)* | 2.06 (1.09-3.90)* |
| SOMI-2 | 3.11 (1.18-8.17)* | 3.07 (1.17-8.07)* | 2.97 (1.13-7.81)* | 3.28 (1.23-8.75)* | 3.32 (1.23-8.95)* | 3.59 (1.35-9.55)* | 3.48 (1.31-9.27)* |
| SOMI-3/4 | 3.17 (1.36-7.40)* | 3.57 (1.53-8.37)* | 3.06 (1.32-7.10)* | 5.06 (2.11-12.15)** | 2.90 (1.24-6.82)* | 4.73 (1.97-11.35)** | 4.81 (1.98-11.69)** |
| PIB Global |  | 1.32 (1.06-1.64)* |  |  |  |  | 1.17 (0.93-1.48) |
| Hippocampal Volume |  |  | 0.76 (0.57-1.02) |  |  |  | 0.98 (0.72-1.35) |
| Entorhinal Thickness |  |  |  | 0.56 (0.44-0.73)*** |  | 0.63 (0.49-0.80)** | 0.66 (0.50-0.88)* |
| Inferior Temporal Thickness |  |  |  | 1.25 (0.95-1.65) |  |  |  |
| Entorhinal FDG |  |  |  |  | 0.62 (0.41-0.92)* | 0.84 (0.63-1.12) | 0.86 (0.64-1.16) |
| Inferior Temporal FDG |  |  |  |  | 1.02 (0.72-1.45) |  |  |
| Hippocampal FDG |  |  |  |  | 1.35 (0.90-2.03) |  |  |

Note: Model 1 only includes SOMI stage and age. Models 2 and 3 also include PIB and adjusted hippocampal volume (HVa), respectively. Models 4 and 5 include, in addition to the core model, either thickness or FDG measures, respectively. Model 6 includes the core model with all entorhinal measures. Model 7 includes the core model, PIB, HVa, and entorhinal measures. †, *, **, and *** indicate p-values less than 0.10, 0.05, 0.01, and 0.001, respectively, after Benjamini-Hochberg correction for multiple testing across 41 statistical tests.

**Supplementary Table 8. Hazard ratios from the Cox Proportional Hazard Models without SOMI.**

| Variable | **1**  **Core** | **2**  **+PIB** | **3**  **+HV** | **4**  **+Thickness** | **5**  **+FDG** | **6**  **+Entorhinal** | **7**  **+PIB+HV**  **+Entorhinal** |
| --- | --- | --- | --- | --- | --- | --- | --- |
| Age | 1.06 (1.01-1.10)† | 1.05 (1.01-1.10) | 1.04 (0.99-1.09) | 1.05 (1.01-1.10) | 1.05 (1.00-1.10) | 1.04 (1.00-1.09) | 1.04 (0.99-1.09) |
| APOE4+ | 1.71 (0.99-2.94) | 1.38 (0.74-2.59) | 1.71 (0.99-2.94) | 1.59 (0.92-2.74) | 1.80 (1.04-3.11) | 1.65 (0.96-2.84) | 1.53 (0.84-2.79) |
| SOMI-1 |  |  |  |  |  |  |  |
| SOMI-2 |  |  |  |  |  |  |  |
| SOMI-3/4 |  |  |  |  |  |  |  |
| PIB Global |  | 1.20 (0.94-1.54) |  |  |  |  | 1.08 (0.84-1.39) |
| Hippocampal Volume |  |  | 0.78 (0.58-1.04) |  |  |  | 0.93 (0.68-1.27) |
| Entorhinal Thickness |  |  |  | 0.65 (0.50-0.83)* |  | 0.70 (0.56-0.89)* | 0.73 (0.57-0.95) |
| Inferior Temporal Thickness |  |  |  | 1.17 (0.89-1.53) |  |  |  |
| Entorhinal FDG |  |  |  |  | 0.62 (0.43-0.89)† | 0.82 (0.62-1.08) | 0.83 (0.63-1.11) |
| Inferior Temporal FDG |  |  |  |  | 1.04 (0.75-1.43) |  |  |
| Hippocampal FDG |  |  |  |  | 1.37 (0.93-2.01) |  |  |

Note: Model 1 only includes age and APOE4 status. Models 2 and 3 also include PIB and adjusted hippocampal volume (HVa), respectively. Models 4 and 5 include, in addition to the core model, either thickness or FDG measures, respectively. Model 6 includes the core model with all entorhinal measures. Model 7 includes the core model, PIB, HVa, and entorhinal measures. †, *, **, and *** indicate p-values less than 0.10, 0.05, 0.01, and 0.001, respectively, after Benjamini-Hochberg correction for multiple testing across 27 statistical tests.
